# Clinic entrance interviews: a new method to assess needs after a sudden impact disaster

**Published:** 2007-10-22

**Authors:** Johan von Schreeb, Niklas Karlsson, Hans Rosling

## Abstract

**Background:**

After a Sudden impact disasters (SID), relief workers and coordinators require information on the size and location of the affected population as well as the character and magnitude of their immediate needs.

**Methods:**

The study was performed in the mountainous Bagh district, a part of the autonomous state of Azad Jammu and Kashmir in Pakistan. Semi-structured interviews were conducted daily at either of the three health centres or the district hospital in which MSF was working.

**Results:**

The MSF facility-based survey results on mortality and injury in Bagh Tehsil corresponded to those from the community-based Army survey. This indicates that regular selection of consecutive arrivals at the entrance of a health facility may provide a fairly geographically representative population sample in a SID context. Our findings suggest that the sample was large enough to provide useful estimates on the main pattern of post-earthquake needs in the study area.

Sudden impact disasters (SIDs) are events such as earthquakes, tidal waves, tropical storms, volcanic eruptions and landslides that cause disruption on such a scale that extraordinary efforts, often involving international assistance, are needed to help the population cope with the aftermath.^1^ After an SID, relief workers and coordinators require information on the size and location of the affected population as well as the character and magnitude of their immediate needs.^2^-^6^ Methodologies for the collection of such information are well described,^3^, ^7^-^9^ but their effectiveness in swiftly identifying and quantifying relevant needs in real life have rarely been documented. Assessments after SIDs are performed in unpredictable contexts, amid destroyed infrastructure and too often without detailed maps or pre-disaster population data.

On 8 October 2005 an earthquake affected an area two-thirds the size of Switzerland, in northern Pakistan and India, with a population of three to four million. On 9 October the Pakistani government requested international assistance, and on October 13 Médecins Sans Frontières Belgium (MSF) started providing relief in Bagh district. Throughout the second month after the earthquake, international relief agencies in Bagh district lacked detailed maps and population data.

To better target its intervention, during the third week after the earthquake MSF piloted a Clinic Entrance Interview (CEI) to collect information on the perceived vital needs and main concerns of the affected population of the Bagh Tehsil area of the Bagh district. The aim of this paper is to assess the utility of the CEI as a quick and simple method for the rapid assessment of needs.

## Methods

The study was performed in the mountainous Bagh district, a part of the autonomous state of Azad Jammu and Kashmir in Pakistan. Inhabited mainly by subsistence farmers, it is among the poorest districts in the country. The under-five mortality is 100 per 1,000 live births.^10^

**Figure 1:**
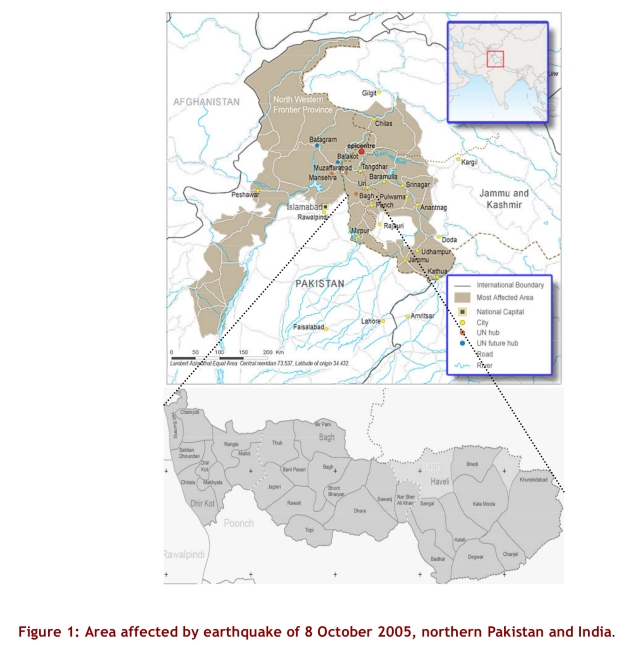
Area affected by earthquake of 8 October 2005, northern Pakistan and India

MSF provided support with staff, material and drugs to the district hospital and a health centre in Bagh city as well to two health centres in the rural community of Bir Pani and Mallot (Figure 1). Services and drugs at the MSF-supported health centres were offered free to all, largely by expatriate doctors and nurses. An average of 50 patients per day were seen in each location. At the district hospital MSF expatriates provided surgical care, while Ministry of Health staff provided inpatient and outpatient care.

Semi-structured interviews were conducted daily at either of the three health centres or the district hospital in which MSF was working. The daily interview site was selected each day by the first author (JVS) on the basis of transport availability. Patients or relatives waiting in the outside queue were selected when passing the entrance to the facility. As soon as an interview was finished, the interviewer would return to the entrance. The next adult person above 18 years of age to enter was selected and invited to be interviewed using a pre-tested form (see Appendix). If several people from one household arrived at once they were asked to decide on the interviewee among themselves. In the survey a household unit was defined as a group of people regularly sharing a meal. We had no written sampling protocol, but the application of the procedures was supervised by the first author on a daily basis. Consent was obtained verbally by asking the selected person whether he or she agreed to be interviewed regarding the situation of his or her household; it was explained that he or she could end the interview at any stage. This survey was done to better target the operational focus of the MSF field work.

The levels of the political and administrative system were Province, District, Tehsil and Union Council. Census data from 1998 were provided by the Population Census Organisation of the Government of Pakistan. The annual population growth rate of Bagh Tehsil was projected at 2.01%.

A local male lawyer was hired to conduct the interviews. He was given a 4-hour briefing on how to select interviewees, conduct the interviews and interpret the responses. Each evening the results of the interviews were read and compiled by the first author. A full analysis of the data was not possible until later, when information on population size and maps with administrative divisions were available. The completed questionnaires were entered into EpiData version 3.1 and exported to EpiData Analysis version 1.1 (EpiData Association, Odense, Denmark) for cross-tabulation and chi-square analysis.

To assess representativeness, the CEI results were later compared with data from an exhaustive post-earthquake house-to-house survey in Bagh district performed by the Pakistani Army, which other humanitarian workers in the region had advised us was the best available. The results of this survey were kindly made available by the Federal Relief Commission. Data on the number of dead and injured people and on the severity of house damage had been collected in Bagh Tehsil between the end of November and the beginning of December.

## Results

**Figure 2:**
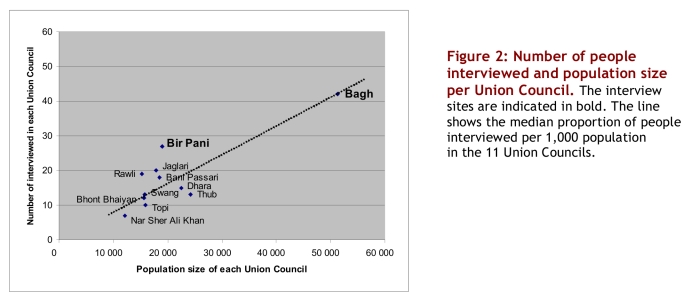
Number of people interviewed and population size per Union Council. The interview sites are indicated in bold. The line shows the median proportion of people interviewed per 1,000 population in the 11 Union Councils

A total of 196 adult respondents (42% women) from an equal number of households were interviewed between 29 October and 19 November 2005. Eight people declined to be interviewed. A majority (124, 63%) of the interviews were conducted at the three health centres; 72 (37%) were conducted at the district hospital. The 196 interviews represented a total of 1,847 pre-earthquake household members, with a range of 2–25 (median 9) persons per household. Both the hospital and the three health centres had respondents from all 11 sub-units (Union Councils) of Bagh Tehsil. Figure 2 shows the relation between the number of interviewees per Union Council and the total population of each Union Council. The number of interviewees per union council inhabitants ranged from 0.5 to 1.4 per 1,000, with a median proportion of 0.8 per 1,000 for all 11 Union Councils.

**Table 1:**
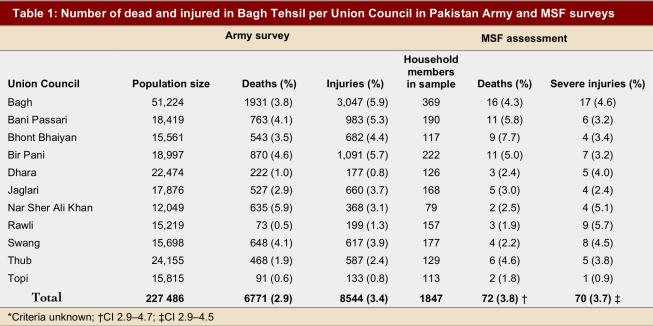
Number of dead and injured in Bagh Tehsil per Union Council in Pakistan Army and MSF surveys

## Injury and death

Of the 1,847 pre-earthquake household members identified through interviews, 72 (3.8%; 95% confidence interval [CI] 2.9–4.7) reportedly died within the first 3 days after the earthquake. The death rate in the Union Councils ranged from 1.8% to 5.8%. A total of 74% of the households reported no deaths, 20% reported one death, 2.5% two deaths, 2.5% three deaths and 1.0% four deaths. The deaths were evenly divided between men (36 persons, 50%) and women (36 persons, 50%); 14 (20%) of the deaths were of children under 5 years of age. There was no statistical (chi-square) association between mortality rate and size of household.

The Army survey found an overall rate of death in Bagh Tehsil of 2.9%, ranging from 0.8% to 5.9% by Union Council. In view of the small sample size per Union Council, the MSF study did not allow disaggregation of mortality rate per Union Council. Standardizing Bagh Tehsil mortality results for population distribution by Union Council did not affect the result. The CEI found that 3.7% (CI 2.9–4.5) of the study population was reported as severely injured, while the Army survey reported 3.4% as injured.

## Vital needs

The main concern expressed by 99% of the respondents was lack of appropriate shelter. A total of 93% of the respondents could not sleep inside their house. Of these, 58% slept in tents, 21% under plastic sheeting, 6% in sheds, 4% had no shelter and 11% used unspecified shelters. Only 8% of the interviewees had access to any form of heating system, while 40% reported not having any blankets in the household. A total of 90% of respondents reported having access to multiple types of food (mainly rice, flour and pulses). Food stocks were estimated to be available for a median of 9 days. Access to water was not expressed as a main concern, and only 5% reported using more time than before the earthquake to collect water. Before the earthquake, 75% of households had used their own latrine, while only 5% used latrines at the time of the interview. Only 30% of respondents reported having soap. A total of 53% of the households had access to health care in their village before the earthquake, while only 16% had access to health services at the time of the interview.

## Discussion

The MSF facility-based survey results on mortality and injury in Bagh Tehsil corresponded to those from the community-based Army survey. This indicates that regular selection of consecutive arrivals at the entrance of a health facility may provide a fairly geographically representative population sample in a SID context. Our findings suggest that the sample was large enough to provide useful estimates on the main pattern of post-earthquake needs in the study area.

The important results were that almost all houses were damaged, whereas food and water were available to the majority of the households. The suggestion that facility-based sampling achieved relatively good geographical representativeness is supported by the narrow range in the proportion of interviewed people per population for the 11 Union Councils. The proportion of interviews from Bir Pani Union Council, where interviews were conducted, was only 75% above the median value for all Union Councils. The explanation for the reasonably good household representativeness from all the subunits in the Tehsil may be that free and reasonably good quality health care attracted a representative sample of the poor and remote population. Another explanation may be that the CEI started 3 weeks after the earthquake, when the worst emergency phase was over and transportation was re-established. In the study period, earthquake-related injuries and their complications represented only 25% of the diagnoses at the three health centres. The most common diagnosis was scabies. The number of new inpatients at the hospital had returned to pre-earthquake levels, and diagnoses for admitted patients were mainly pneumonia, other infectious diseases, cardiac failure and non-earthquake-related trauma.

### Study limitations

One limitation of this study was that we were unable to document the validity of our results on the vital needs of the studied population. Nevertheless, the relatively good geographical representativeness encouraged MSF to use the results for relief planning. Another limitation was that the questionnaire had too many questions. Although semi-quantitative in nature, it included some questions of a more qualitative character. However, the results from such open-ended questions require triangulation with other qualitative methods. Such a survey design would yield a better understanding of the perceived needs and concerns of the affected population.

Our sampling framework had several limitations. One was that site selection was not systematic, being limited by transport availability. Further, subject selection was done by convenience. We had planned a systematic sampling of every seventh person to arrive at the health facility, but this turned out to be impossible given available resources. This type of sampling of an ill-defined group of visitors should never be considered as an alternative to an established, representative sample of the population living in a defined area, but in the context of an SID, when urgent assessment is needed and resources are limited, it appears to suffice. It is noteworthy that it took more than two months for international agencies to obtain access to detailed maps of Bagh Tehsil with administrative divisions and pre-earthquake population data.

The concepts and methodologies behind the CEI can easily be expanded to include sampling and interviews at other locations that attract the population in the affected area. The CEI used health facilities for both sampling and interviews. Although it might be tempting to increase the number of interviews at each site, this will not reduce bias. Instead, it seems advisable to increase the type and number of interview sites to include other “service” locations, such as water or food distribution sites or even the market, accepting that the same household may be interviewed at more than one location. Methods to deal with other forms of potential selection bias need further study. Further, more work is needed to determine the optimal number and type of questions.

The results from service-site surveys may be rapidly analysed and made immediately available to target humanitarian assistance based on the prioritized needs and location of the affected population. Service-site surveys should be started by specifically designated staff in parallel with relief activities, and should be repeated regularly to allow monitoring of changing needs until surveys with representative samples are performed.

The CEI can yield timely data and semi-quantitative estimates of humanitarian needs after SIDs and thus may be a useful decision-making tool. It is a fast and effective method to obtain more and better information with reasonable representativeness while waiting for results of population-based surveys. We propose that the service-site survey concept be further developed and tested.
