# Supplementary material for: Clinic entrance interviews: a new method to assess needs after a sudden impact disaster
Source: Open Med. 2007 Oct 22;1(3):e153–9. (PMC3113225)
Supplement: Supplementary file 1 [file OpenMed-01-e153-s001.pdf]

Appendix: Clinic entrance interview form.

<table>
<tr><td colspan="4">Date:</td><td colspan="3">Filled in by:
In OPD/ hospital / village</td></tr>
<tr><td colspan="4">Informant: Male Female</td><td colspan="3">Head of Household: Male
Female (if widow post-EQ)</td></tr>
<tr><td colspan="4">VILLAGE:
Union Council: .</td><td colspan="3">Teshil:
GPS coordinate:</td></tr>
<tr><td colspan="7">Household size pre-EQ:
Females: Males: Children under 5:</td></tr>
<tr><td colspan="7">Dead people How many: Male: Female: Ages:</td></tr>
<tr><td colspan="7">House damaged Y / N Can be used for sleeping Y / N</td></tr>
<tr><td>INJURIES</td><td>Total</td><td>Male</td><td>Female</td><td>< 15 years</td><td># referred</td><td>Remarks</td></tr>
<tr><td>Severely injured</td><td></td><td></td><td></td><td></td><td></td><td></td></tr>
<tr><td colspan="7">Referred to where?</td></tr>
<tr><td colspan="7">HEALTH & HEALTH FACILITIES</td></tr>
<tr><td colspan="3">pre-EQ: Health care available in village:
Y / N Type:</td><td colspan="4">If no, distance to the closest: Location:
Type:</td></tr>
<tr><td colspan="3">post-EQ Health care available in village:
Y / N Type:</td><td colspan="4">Other medical assistance post-EQ (e.g., private MDs, army, NGOs)</td></tr>
<tr><td colspan="7">OTHER VITAL POST-EQ NEEDS</td></tr>
<tr><td colspan="4">WATER SOURCE(S)
pre-EQ___________ post-EQ________
Distance in km/time: Distance in km/time:
Transportation: Transportation:
Material for carriage: Material for carriage:
Chlorination: Y / N Chlorination: Y / N
Freezing: Y / N Freezing: Y / N
EXCRETA DISPOSAL WASTE MANAGEMENT:
pre-EQ_____________ HYGEINE
post-EQ____________ soap available Y / N

BLANKETS per household:
ELECTRICITY Y / N PHONE (land line) Y / N</td><td colspan="3">FOOD SECURITY Sort of food:

Source (e.g. market, agriculture, life stock, distribution):

Sufficient until when:
Plans after:

FUEL FOR COOKING:
COOKING SET: Y / N

SHELTER TYPE: Winterized Y / N
HEATING SYSTEM: Y / N Source:</td></tr>
<tr><td colspan="7">DISTRIBUTION / ASSISTANCE performed by:</td></tr>
<tr><td colspan="7">Where did your family spend last winter? In the village / Another place if yes, where?
Where are your family planning to spend this winter? Stay in the village / Migrate if yes, to where?
What is the major concern / need for the moment?
Where did the woman in your family give birth last time?
What did you do when a child in the family had fever last time?</td></tr>
<tr><td colspan="7">SUMMARY OF NEEDS & ADDITIONAL COMMENTS</td></tr>
</table>
